# Stimulus generalization and return of fear in C57BL/6J mice

**DOI:** 10.3389/fnbeh.2012.00041

**Published:** 2012-07-16

**Authors:** Yannick Boddez, Zsuzsanna Callaerts-Vegh, Bram Vervliet, Frank Baeyens, Rudi D'Hooge, Dirk Hermans, Tom Beckers

**Affiliations:** ^1^Department of Psychology, KU LeuvenLeuven, Belgium; ^2^Department of Psychology, University of AmsterdamAmsterdam, Netherlands

**Keywords:** generalization, extinction, exposure therapy, return of fear, relapse, C57BL/6J mice

## Abstract

Return of fear following successful exposure therapy is a common problem. More insight into the characteristics of extinction learning is crucial in enhancing the efficiency of therapeutic interventions. In particular, understanding the mechanisms that underlie the generalization of extinction learning to other discrete stimuli is indispensable. Presently, little is known about the molecular and genetic mechanisms underlying this phenomenon. In this study, we attempt to develop a new conditioning protocol to study return of fear, caused by a stimulus change after extinction, in the most commonly used mouse strain of behavioral genetics, C57BL/6J. Perceptual changes to an auditory fear conditioned stimulus led to return of fear after initially successful fear-reduction, relative to appropriate control treatment. We argue that this protocol will be a useful tool to unravel the neurobiological underpinnings that regulate generalization of extinction and return of fear. Key questions for future research include the identification of crucial brain structures, neurotransmitters and signaling pathways that underly this behavioral phenomenon. Arguably, such research will open up new perspectives for neurobiological therapy augmentation.

## Introduction

Exposure-based therapy is a highly effective treatment for several anxiety disorders. The general principle in this kind of treatment is to repeatedly expose patients to their object of fear. Despite the initial efficacy of this approach, there is substantial evidence in the clinical literature that not all patients remain free of symptoms at follow-up. For some patients, there is a return of fear (i.e., relapse; Rachman, 1989; Craske et al., 2006).

It is essential to develop animal models of relapse in order to reveal its neurobiological mechanisms and to optimize therapeutic interventions. Mice are subjects of preference in molecular and genetic memory research, mainly because there is a substantial body of knowledge concerning mouse genetics (Laxmi et al., 2003; Waddell et al., 2004). The goal of the present study is to develop a behavioral conditioning protocol for a hitherto largely ignored candidate pathway to return of fear in a mouse model. We employed the most commonly used mouse strain of behavioral genetics, C57BL/6J.

The standard fear conditioning procedure entails the contingent presentation of a conditioned stimulus (CS; e.g., a tone) with an aversive unconditioned stimulus (US; e.g., a foot shock). The resulting fear response to the CS can be extinguished by repeated presentations of the CS without the US, providing an experimental analogue of exposure treatment. The dominant protocols to obtain return of fear in the laboratory are the insertion of a context switch, unsignaled US presentations or a retention interval between extinction training and testing (i.e., respectively renewal, reinstatement, and spontaneous recovery; (e.g., Bouton, 2002; Waddell et al., 2004; Hermans et al., 2006). These laboratory protocols have been connected to return of fear in clinical practice. For example, it is argued that a switch from the therapeutic context to the home context can be a sufficient condition to elicit return of fear following successful exposure treatment (i.e., renewal; e.g., Bouton, 2002).

Renewal, reinstatement and spontaneous recovery protocols successfully model how post-extinction events can interfere with the extinction of fear originally conditioned to the CS. In clinical practice, exposure to the original CS is, however, often impossible, like when the original CS cannot be identified or when the CS cannot be presented because of practical or ethical reasons. In such cases, the clinician will often expose to generalization stimuli (GSs; e.g., other dogs than the one involved in a biting incident). A number of learning theories suggest that exposure to such GS may constitute an alternative pathway to relapse when the patient is again confronted with the original CS (or a different GS) following treatment. These theories stipulate that the degree of generalization is a function of the elements that the CS and the GS hold in common (Rescorla, 1976; Pearce, 1987). Exposure to a GS will reduce the associative strength between these common elements and the US, but upon confrontation with the original CS, the nonextinguished elements unique to the CS will come into play, possibly leading to a return of fear. In an attempt to model this candidate pathway to relapse, in the present study we used a conditioning protocol that entailed exposure to a GS followed by testing of the original CS. Interestingly, according to the common-element analysis, fear extinguished by exposure to the original CS should not as easily return upon confrontation with a GS: Fear responding to a GS after extinction of the original CS will solely be determined by the strength of the common elements -note that the elements unique to the GS have never been paired with the US- and should thus be similar to fear responding seen at the end of extinction.

A behavioral protocol for this alternative candidate pathway to return of fear is currently lacking. The development of such protocol may help to further identify key neurobiological mechanisms underlying return of fear in future research. This is particularly important because different pathways to return of fear may recruit from different neural circuits.

Table 1 summarizes the timeline of the procedure. There were two experimental groups (AbBa and AbAb) and one control group (AaAa). Following identical acquisition training, subjects in group AaAa were tested with the original CS, whereas subjects in groups AbAb and AbBa were tested with a GS. This test allowed evaluating whether acquisition generalized from the CS to the GS, which is a prerequisite for studying subsequent extinction learning. Afterwards, groups AaAa and AbAb received extinction training using the original CS, whereas group AbBa received extinction training with the GS. We then implemented a stimulus change in groups AbAb and AbBa in order to investigate to what extent extinction learning with a GS would generalize to the original CS (AbBa group) and vice versa (AbAb group). We anticipated return of fear in the AbBa group, but not in the AbAb group, both relative to the AaAa control group.

**Table 1:** **Timeline of the training procedure**.

	Day 1	Day 2	Day 3	Days 4–28	Day 29
	↓	↓	↓	↓	↓
	Context pre-exposure	Acquisition	Generalization of acquisition	Extinction	Generalization of extinction
Group: AaAa		2A+	2A−	3A−	2A−
Group: AbAb		2A+	2B−	3A−	2B−
Group: AbBa		2A+	2B−	3B−	2A−

See main text for details.

Note. A: Conditioned Stimulus and B: Generalization Stimulus. Gray fill color indicates context 1 and absence of fill color indicates context 2.

Numerals indicate number of trials.

## Materials and methods

Subjects were 24 female C57BL/6JRj mice (Charles River, France) and were 84 days old at the start of the experiment. They were housed in two cages of 12 animals each in a temperature and humidity controlled animalium (12 h/12 h light-dark cycle, 22°C), where they had free access to food and water. Training was conducted during the light phase of the activity cycle. On each day, mice were trained sequentially in a single fear conditioning setup. Animals were removed from their home cage and were placed in the conditioning apparatus about 60 s later. At the end of a session, mice were immediately returned to their home cage. To avoid covariation between the home cages and the experimental conditions, we randomly assigned one-third of the subjects in each home cage to each of the three experimental conditions. Note that this approach ensures that differences between conditions cannot be attributed to differences between cages, but does not prevent that mice might notice fear reactions in previously trained mice returned to the home cage. The training protocol has been reviewed and approved by the animal experiments committee of the University of Leuven (Belgium) and was carried out in accordance with the European Community Council Directive (86/609/EEC).

Animals were trained in a fear conditioning setup with a weight transducer system that allows for automated movement detection (Startle and fear combined system, Panlab, SL, Barcelona, Spain). The animal compartment of this setup is made of black methacrylate walls and a transparent front door, is soundproof and measures 250 × 250 × 250 mm. A tone (70 dB, 6000 Hz, 30 s) and a white noise (70 dB, 30 s) served as CS and GS, completely counterbalanced, and were administered through a built-in loudspeaker. A constant current footshock (0.3 mA, 2 s) was delivered through the grid floor and served as US. Before the commencement of the study, we determined the activity threshold under which the animal's behavior could be considered as freezing, defined as the absence of all movement except of breathing. The percentage of time below this activity threshold during stimulus presentation, CS or GS, served as dependent variable. Acquisition training took place in context 1: Paper (Coloraction Deep Green A4 Color paper 80 g/m^2^, Antalis, Austria) was attached to the walls of the animal compartment and the grid floor was cleaned with 70% ethyl alcohol to provide a background odor. The other training and test phases were carried out in context 2. There was no paper attached to the walls of the animal compartment in context 2 and instead of ethyl alcohol, a cotton ball with a peppermint solution was placed in the chamber in order to provide the background odor. Stimulus presentation, data acquisition and data reduction were carried out with computer software from Panlab (Freezing, Panlab, SL, Barcelona, Spain).

The training procedure consisted of five phases: a pre-exposure phase, an acquisition phase, a first generalization test, an extinction phase and a second generalization test. Subjects received pre-exposure, extinction and testing in a context (context 2) different from the acquisition context (context 1) to prevent contextual fear from masking differences in fear elicited by the CS and GS during testing and extinction. During the pre-exposure session, carried out on the first day of the experiment, all subjects were exposed to context 2 for 15 min. The pre-exposure was included in the design in order to enhance discrimination between the acquisition context, context 1, and the extinction and the generalization test context, context 2. On the second day, we proceeded with acquisition training. After 2 min of exploration, the first CS-US pairing was presented. After another 20 min, a second CS-US pairing was delivered. The 2 s US co-terminated with the CS both times. Before the end of the session, subjects were allowed to explore the context for another min. On the third day, subjects in group AaAa were tested with the original CS, whereas subjects in the groups AbAb and AbBa were tested with the (novel) GS. After 1 min of exploration, the 30 s CS or GS was presented, followed by a 1 min interval, which was in turn followed by a second CS or GS presentation. The session ended with a 90 s exploration period. On days 4–28, extinction treatment was administered. On each day, subjects in the AaAa and AbAb groups received 3 unreinforced presentations of the CS, whereas subjects in the AbBa group received 3 unreinforced presentations of the GS. The discrete stimuli were again presented for 30 s and there was a 90 s interval between stimulus presentations. Each session started with 90 s of exploration and ended with 60 s of exploration In total, this resulted in an extinction session of seven min, which we considered a manageable length of time for a training session that was repeated over multiple days. On day 29, subjects in the AaAa and AbBa groups were tested with the original CS, whereas subjects in the AbAb group were tested with the GS. As during the first test phase, a first 30 s CS or GS presentation was followed by a 60 s interval, which was in turn followed by a second CS or GS presentation. The session started and ended with 90 s of exploration.

## Results

We applied data reduction to extinction and test data. More precisely, we averaged the percentage of freezing over the two stimulus presentations of the test sessions and over the three stimulus presentations of the extinction sessions. Acquisition data were not reduced, because there were only two acquisition trials and we were interested in a potential increase in freezing from the first to the second trial.

**Figure 1:**
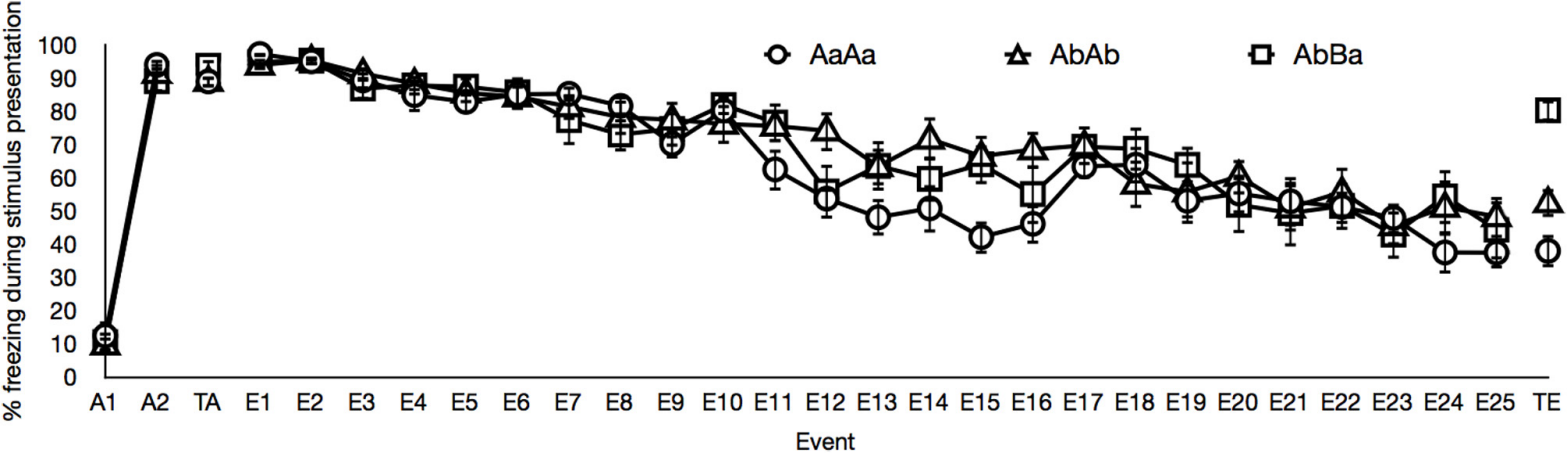
**Mean percentages of freezing for events of interest in the AaAa group (circles), in the AbAb group (triangles) and in the AbBa group (squares).** The graph displays freezing during both acquisition trials (A1–A2), freezing averaged over the two generalization of acquisition test trials (TA), freezing averaged over the three extinction trials on all days of extinction training (E1–E25) and freezing averaged over the two generalization of extinction test trials (TE). Error bars represent the standard error of the means. See main text for details.

To exclude the influence of contextual fear and unspecific effects that are not related to the CS or GS under investigation (e.g., sensitization or habituation), we also analyzed the data using difference scores of each stimulus and its pre-stimulus interval of the same length. The results of the analyses on these difference scores are not reported, because these results did not differ from the analyses on the absolute percentages of freezing. The data of two sessions were lost because of technical error: The acquisition data of a subject belonging to the AbBa group and the extinction data on day 3 of another subject belonging to the AbBa group. Figure 1 depicts the percentages of freezing over the course of training, by group.

With respect to acquisition training, Figure 1 demonstrates that the intensity of the fear response was higher to the second CS presentation than to the first CS presentation in all groups, indicative of an acquisition effect. A mixed Group by Time (Acquisition Trial 1, Acquisition Trial 2) ANOVA indeed revealed a main effect of time, [*F*_(1, 20)_ = 698.63, *p* < 0.01]. This acquisition effect did not differ between groups, [*F*_(1, 20)_ < 1].

To assess generalization of acquisition, we compared the percentage of freezing for the last acquisition trial with the percentage of freezing averaged over the two test trials. The AaAa group was tested with the original CS, whereas the AbAb and AbBa groups were tested with the generalization stimulus. A mixed Group by Time ANOVA revealed no decrease in fear responding from the end of acquisition to the generalization test, [*F*_(1, 20)_ < 1], nor an interaction with group, [*F*_(1, 20)_ < 1]. This suggests that responding to the GS did not differ from responding to the CS, suggesting generalization of acquisition performance.

Figure 1 shows the decrease in fear responding to the CS (groups AaAa and AbAb) and to the GS (group AbBa) from the first day of extinction to the last day of extinction. A mixed Group by Time (extinction day 1 until extinction day 25) ANOVA revealed a main effect of time, [*F*_(6.55, 137.47)_ = 47.52, *p* < 0.01]. We observed no significant effect of group and no group × time interaction, respectively [*F*_(2, 21)_ < 1 and *F*_(13.09, 137.47)_ = 1.43, *p* = 0.15]. This suggests that the course of extinction did not differ between groups.

To assess generalization of extinction, we compared the mean percentage of freezing over the two final test trials to the mean percentage of freezing during stimulus presentations on the last day of extinction. The resulting Group by Time ANOVA revealed main effects of group, [*F*_(2, 21)_ = 6.42, *p* < 0.01], and time, [*F*_(1, 21)_ = 9.08, *p* < 0.01], and a significant group × time interaction, [*F*_(2, 21)_ = 6.22, *p* < 0.01]. Planned comparisons revealed that the AbBa group displayed a significant increase in freezing from the end of extinction to the generalization test, [*F*_(1, 7)_ = 8.38, *p* < 0.05], which was absent in the AaAa and AbAb groups, both *Fs* < 1. Accordingly, the increase observed in the AbBa group differed from the pattern observed in the AaAa group, [*F*_(1, 14)_ = 7.62, *p* < 0.05], and the AbAb group, [*F*_(1, 14)_ = 5.83, *p* < 0.05]. The pattern observed in the latter two groups did not differ, [*F*_(1, 14)_ < 1].

## Discussion

In this study, we aimed to develop a new training protocol for studying relapse following successful fear-reduction treatment in C57/BL6J mice. Subjects readily acquired fear to the CS in the acquisition phase. The generalization of acquisition test showed that the GS came to elicit a strong fear response as well. Following successful extinction in all groups, we implemented a stimulus change in the AbAb group and in the AbBa group to test generalization of extinction performance. The AbBa group displayed a large generalization decrement, evidenced by a steep increase in fear responding upon the stimulus change after extinction. This demonstrates that the behavioral effects of extinction learning with a GS do not generalize to the original CS and this procedure therefore provides a novel method for studying return of fear in C57BL/6J mice. We did not observe generalization decrement in the AbAb group, which suggests that the effects of extinction learning with an original CS do generalize to other stimuli. The same asymmetry has been found in humans, the species that would ultimately benefit from this research, vowing for the translational validity of the present training protocol for studying relapse in C57/BL6J mice (Vervliet et al., 2004). The same pattern of results was observed using difference scores of each stimulus and its pre-stimulus interval of the same length, which indicates that measured lack of movement can indeed be attributed to fear-induced freezing behavior rather than to general inactivity.

It is important to note that the high responding to the GS in groups AbAb and AbBa during the generalization of acquisition test cannot be due to neophobia. The CS and the GS were completely counterbalanced and freezing during the first stimulus presentation, in the first acquisition trial, was consistently low (Figure 1). The gradual decline of freezing across extinction sessions is relatively slow compared to previous studies (e.g., Stiedl et al., 1999; Siegmund et al., 2005), which might be due to enhanced fear learning in some of the animals through stress transmitted by their conspecifics during group housing in the home cage.

The lack of generalization decrement following extinction in the AbAb group could be criticized on the basis of subjects in the AbAb group receiving two unreinforced presentations of the GS during the generalization of acquisition test. In principle, one could attribute the low fear responding during the generalization of extinction test to these preceding unreinforced trials. Two extinction trials are however substantially insufficient to produce an extinction effect in C57BL/6J mice. This is illustrated by the amount of freezing during the first day of extinction in the AaAa group and the AbBa group. As illustrated in Figure 1, freezing in the AaAa group and in the AbBa group to respectively the CS and the GS was not lower on the first day of extinction than during the generalization of acquisition test, despite receiving two unreinforced presentations of these stimuli during the generalization of acquisition test. The lack of generalization decrement following extinction in the AbAb group is moreover in line with the weak nature of AAB renewal in studies on contextual control over extinction (Bouton, 2002). AAB renewal is described as a renewal of conditioned responding when going to a new context B, after acquisition and extinction training in context A. The AAB renewal effect is generally weak (as compared to ABA renewal; Bouton, 2002). Further it should also be noted that only female mice were used, which may provide a different result than a mixed sex subject base or a male subject base and that C57BL/6J mice may show behavioral differences from other C57BL/6 substrains (Siegmund et al., 2005).

The development of behavioral methodology is an important first step in behavioral neuroscience. The present training protocol may serve as a powerful method to study relapse, just like the contextual dependency of extinction has been used as a model to study relapse (Radulovic et al., 1998; Bouton, 2002; Waddell et al., 2004). Over the last years, anatomical, physiological and genetic studies have identified important aspects of the neural structures and the neurochemical processes that appear to be involved in fear acquisition and fear extinction (Hefner et al., 2008; Milad and Quirk, 2012; Tronson et al., 2012). Less is known about stimulus generalization processes in extinction despite an increased understanding of generalization processes at the behavioral level (Ghirlanda and Enquist, 2003) and despite the presumed importance of generalization processes in the development (Lissek et al., 2010; Lenaert et al., 2011) and treatment (Vervliet et al., 2004, 2005) of anxiety disorders. In future experiments, the present training protocol may allow unraveling the neurobiological underpinnings of stimulus generalization in extinction. Key question for future research include the identification of crucial brain structures, neurotransmitters and signaling pathways that underly this behavioral phenomenon. Arguably, such research will open up new perspectives for neurobiological therapy augmentation.

### Conflict of interest statement

The authors declare that the research was conducted in the absence of any commercial or financial relationships that could be construed as a potential conflict of interest.
